# Street Cleansing

**Published:** 1900-04-21

**Authors:** 


### Street Cleansing.

There seems to be much reason to hope, and even, thanks to the prevailing trend of public opinion, to believe, that the new municipalities about to be created in the metropolis will be largely composed of residents who will be likely to bestir themselves for the systematic improvement of local management in the directions in which its present defects are most apparent to all whose business or pleasure calls upon them to traverse the streets of London. It would even appear that the London County Council, turning away for the moment from "vast" enterprises, and leaving the inhabitants of Wales to possess their valleys and their rainfall in peace, is about to show that it rivals the trunk of an elephant in its capacity to deal alike with small things and with great by undertaking to organise and direct a conference of municipal authorities, for the purpose of arriving at some general agreement as to the reforms which are most necessary with reference to their duties, and as to the way in which they may most successfully be carried into effect. Among these there seems to be a general consent that a better and more effective method of street cleansing and of street watering is urgently required, and that this is among the first things to which attention should be directed. In this respect, as far as its main thoroughfares are concerned, the parish of St. Marylebone is, probably, by far the worst treated in London; at any rate it is most in evidence, although there are comparatively small streets, running out of main thoroughfares in such parishes as Kennington or Lambeth, which might even deprive Harley Street and Queen Anne Street of their pre-eminence in dirt and untidiness. The residents in Kennington and Lambeth may, perhaps, be more liberal distributors of orange peel, waste paper, and dead cats than the residents in St. Marylebone; but the vestry of the latter parish has hitherto "taken the cake" in the direction of liberality in the manufacture of mud, and of neglect or tardiness with regard to its removal. It would be difficult to say, indeed, when the main streets of Marylebone are at their worst, whether at the time when an abundance of rain or free watering has converted the carriage-way into a Slough of Despond, not to be crossed with impunity by pedestrians, or when a spell of dry weather, perhaps aided by a north-east wind, has converted the mud into dust, which is carried in clouds into every open window, and into the lungs of every passenger. In the form of mud, the filth is perhaps no more than a nuisance; but, in the form of dust, it unquestionably becomes an insanitary influence of great importance.

Many years ago, we believe in 1872, the difficulties of street-watering were practically overcome, for the single summer, in the parish of Paddington. An ingenious gentleman, named Cooper, pointed out that if the streets were wetted with a solution of certain deliquescent neutral chlorides, instead of with pure water, a very much smaller amount of liquid would be required. He took out a patent for the purpose, and the Vestry of Paddingdon gave his system a trial, and proved it to be brilliantly successful. The chlorides used were those of calcium and sodium, and the solution was sprinkled from a water-cart in the usual way. As the water evaporated, the deposited salts absorbed more from the atmosphere, and the tendency of the surface to dry was greatly diminished. When actual drying occurred, the dry surface became covered with a sort of flake, which broke into pieces under traffic, but which had scarcely any tendency to pulverisation, and yielded scarcely any dust. The solution was innocuous highly antiseptic, and entirely stopped the liberation of ammonia from horse-droppings, with the result that, on a dry Sunday, such a thoroughfare as the Harrow Road presented a clean, hard surface, free, not only from dust, but also from the ordinary Sunday smell of less favoured parts of the metropolis. The shopkeepers in the district were unanimous in their praise of the method, and in their testimony to the degree in which their goods were preserved by it from the injury which dust commonly inflicted upon them. The inventor, however, became financially embarrassed, there were various claims to the proprietorship of his patents, and the vestry were hindered, by difficulties hence arising, from continuing or extending the application of the method, which, although its efficacy was only shown upon macadam, would certainly be still more applicable to wood. The patent or patents must long ago have expired, and there seems no valid reason why the invention should not again be employed by some of the new municipalities. What its effects on boots might be we cannot say, but so far as relates to dust and mud no one who has seen it in operation can doubt its efficacy.

